# Definition of customer requirements in big data using word vectors and affinity propagation clustering

**DOI:** 10.1177/09544089211001776

**Published:** 2021-03-16

**Authors:** Yanlin Shi, Qingjin Peng

**Affiliations:** Department of Mechanical Engineering, 8664 University of Manitoba, Manitoba, Canada

**Keywords:** Product design, customer requirement, big data, online review, word vector, web crawling

## Abstract

Customer requirements (CRs) have a significant impact on product design. The existing methods of defining CRs, such as customer surveys and expert evaluations, are time-consuming, inaccurate and subjective. This paper proposes an automatic CRs definition method based on online customer product reviews using the big data analysis. Word vectors are defined using a continuous bag of words (CBOW) model. Online customer reviews are searched by a crawling method and filtered by the parts of speech and frequency of words. Filtered words are then clustered into groups by an affinity propagation (AP) clustering method based on trained word vectors. Exemplars in each clustering group are finally used to define CRs. The proposed method is verified by case studies of defining CRs for product design. Results show that the proposed method has better performance to determine CRs compared to existing CRs definition methods.

## Introduction

The quality of product design highly depends on effective collections of customer requirements (CRs) to decide function requirements (FRs).^1^ CRs are normally used in product design process for clarifying customer needs to guide designers to decide functions and structures of the product.^2^ Accurate CRs can improve competitiveness of products in the market by removing unnecessary functions to reduce manufacturing cost and add necessary functions for improving customer satisfactions.

There are two steps for defining CRs including collection of customers’ comments by customer surveys and definition of CRs by customers’ comments analysis. However, the existing customer survey methods such as focus groups and web-based survey methods are time-consuming and subjective because these methods may take days to interview different customers for a particular product, which is costly and time-consuming.^3^ The existing customers’ comments analysis methods such as affinity diagram method and subjective clustering method are also time-consuming and inaccurate.^4^ Because similarity of customers’ comments in the customers’ comments analysis methods are defined by evaluation of experts and designers manually, which is too subjective.

With the increased number of customers on online shopping, a huge amount of customer reviews for products are available on webpages of online shops such as Amazon.com, BestBuy.com, and Alibaba.com.^5^ These online product reviews provide sufficient information to understand performances of the product based on customer comments to improve design of products. As a huge amount of customer reviews are available online, finding CRs from online customer reviews is an efficient way compared to customer surveys and expert evaluations. However, there is not an existing method to effectively collect online customer review data and transfer the data into specific CRs for a product.

Crawling methods such as focused crawling and deep web crawling methods are widely used for collecting online data. Data mining methods such as clustering, classification and association rule learning are commonly used to extract useful information and knowledge from big data. Using crawling and data mining methods, online customer reviews and sales profit can be collected to find CRs for product improvement. Therefore, a CRs definition method is proposed in this paper using web crawling and data mining methods based on online customer reviews.

To improve the accuracy of CRs definitions in product design, the focused crawling method is used to collect related articles of products. The CMBO method is used to define word vectors in the specific field of related products. Words of customer review comments are filtered based on the content and frequency of words. Using the trained word vectors, filtered words from online customer reviews are clustered into groups by the AP clustering method. Each exemplar in a group is used to define a CR.

Following parts of the paper are organized as follows. Literature review of the related research is described in the following section. A new CRs automatic definition method is proposed in the next section. Further section discusses two case studies of CRs definitions for upper limb rehabilitation devices and mini-fridges. Research conclusion and further work are described in the last section.

## Literature review

### Existing methods of CRs definitions

Existing methods of CRs definitions including affinity diagram method and subjective clustering method can transfer collected comments from customer surveys into CRs. The affinity diagram method generates CRs by classifying collected customer comments from questionnaires into different levels of the dendrogram using a hierarchical clustering algorithm. The similar customer comments are combined at the lowest level of the dendrogram, which can summary similar customer comments to generate a CR. Wu et al proposed a dendrogram with three levels to define CRs of the baby stroller based on customer comments using the affinity diagram method.^6^ Song et al. combined the affinity diagram method with an analytic hierarchy process (AHP) method for pair-wise comparisons of collected comments to improve the accuracy for the CRs definition in design of industrial products.^7^ The affinity diagram method requires the comparison of similarity for collected comments by experts or designers manually, which is costly and time-consuming. In addition, it is difficult to define different levels of the dendrogram accurately because the similarity of customer comments is defined by designers subjectively.

The subjective clustering method determines CRs based on grouping of customer comments from the expert evaluation using a similarity matrix. Grouping results of customer comments defined by several experts are used in constructing the similarity matrix to cluster similar customer comments and define a CR for each cluster. For example, Takai et al improved the subjective clustering method to determine CRs from customer comments using a co-occurrence matrix.^8^ The subjective clustering method requires a similarity matrix built by the expert evaluation for balancing conflict comments from different customers to decide CRs, which is also time-consuming and inaccurate.

In summary, the existing methods in defining CRs have following three problems. 1) Data are analyzed manually in these methods, which is time-consuming. 2) CRs are defined by a limited number of customers or experts, which reduces the accuracy of CRs. 3) There is not an accurate and efficient method for the data analysis to include all important CRs.

### Existing methods of words meaning definitions

The meaning of words is normally represented by word vectors based on similarity of words. There are two kinds of methods for defining word vectors including the CBOW model and Skip-gram model. Both models learn the underlying relationship between words and their contexts.

The CBOW model takes the context of each word as the input and tries to predict the word corresponding to the context. Enríquez et al proposed a method to improve accuracy of word vectors for the opinion classification by combing the CBOW model and a voting system.^9^ Wang et al proposed an improved CBOW model by considering weights of relative positions of adjacent words in the input layer.^10^ The CBOW model has a high training speed and a high quality of the representation for general words with a high frequency in dataset.

The Skip-gram model can find the most related words for a given dataset, which can predict the context word for a given target word. Song et al proposed a directional Skip-gram by explicitly distinguishing left and right contexts in the word prediction.^11^ Liu et al proposed an improved a multi-prototype Skip-Gram model to learn multiple embedding per word type by the interaction between words and topics simultaneously.^12^ The Skip-gram model works better for defining word vectors for small amounts of data and represents rare words very well.

Comparing with the Skip-gram model, the CBOW model can better define word vectors for describing meaning of general words in the proposed method. Most customers provide online comments using general words rather than uncommon words. Therefore, CBOW is selected to define word vectors in the proposed method.

### Definition of CRs using clustering methods

Clustering methods combine similar words of customer reviews to a special group for CRs. K-mean clustering, AP clustering, and nonlinear clustering methods are commonly used for text clustering.

The K-mean clustering is the most popular method for clustering text into words clusters. Alghamdi et al proposed a document representation model using the Bayesian vectorisation along with k-means to improve accuracy and efficiency of text clustering for high-dimensional data.^13^ Kasthuri analyzed the performance of information retrieval and extraction for Tamil language based on the iterative affix stripping stemmer using the K-mean clustering.^14^ The K-mean clustering method is efficient for clustering words into a few groups.

The AP clustering method is based on the concept of “message passing” between points of a database. Guan et al proposed a semi-supervised text clustering algorithm using a seed construction method and AP clustering to improve accuracy and clustering execution time.^15^ Shrivastava et al. proposed a phrase affinity clustering algorithm for a text document clustering based on phrase similarity using affinity propagation to improve the clustering accuracy.^16^ The AP clustering method is accurate for word clustering because it can cluster asymmetric data.

The nonlinear clustering method can cluster words into several groups by considering the uncertainty of word meaning.^17^ Yu et al. proposed bursting patterns for the nonlinear characters of vectors using the fifth order polynomial stiffness nonlinearity, which can improve the accuracy of nonlinear clustering methods.^18^ Wang et al. improved the nonlinear clustering method by mapping the data set into a high-dimensional space to determine the word classification based on similarity of word meaning.^19^

Comparing with other clustering methods, the AP clustering has a better performance in defining CRs from online customer review comments as it can cluster collected words that are not symmetric or do not satisfy the triangle inequality. Its clustering results are also deterministic and do not require the initialization and pre-assigned number of clusters. In addition, it can find an exemplar to represent the meaning of all the words in a cluster. Therefore, the AP clustering method is selected in the proposed CRs definition method.

## Proposed method of CRs definition

**Figure 1.**
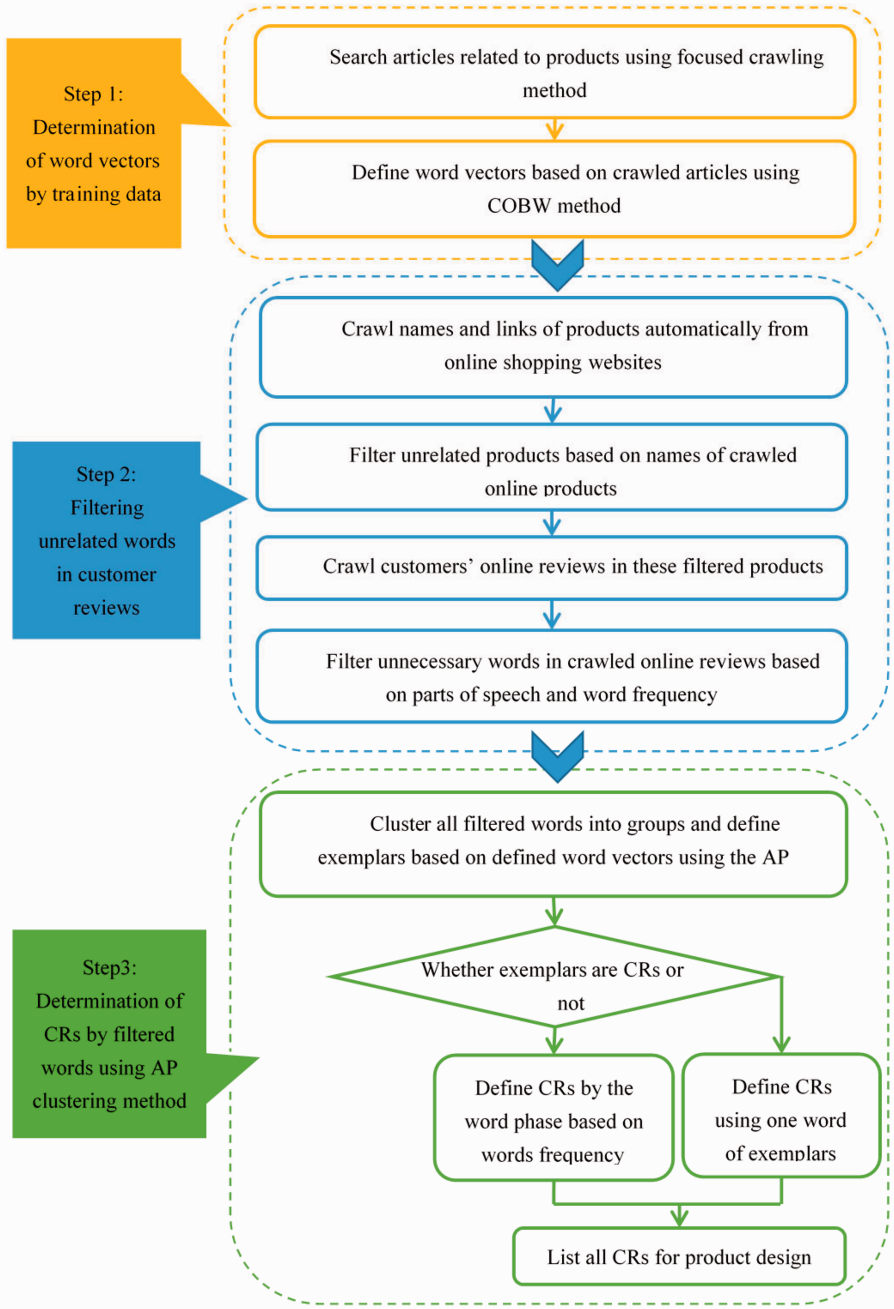
Proposed CRs definition method.

Raw data required for the proposed method are online articles and customer reviews of product. A flow chart of the proposed method is shown in Figure 1.

### Determination of word vectors using collected training data

**Figure 2.**
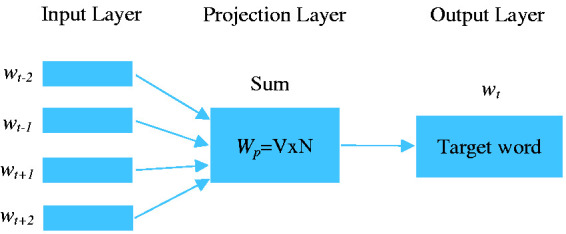
Word vectors definition by CBOW.

For reducing the influence of polysemy, related articles of a target product are searched using the focused crawling method to form a dataset. Word vectors can be defined by training a set of fixed-length vectors based on a large corpus of text. Each word is represented by a point. All collected points can be trained to improve accuracy of meaning of words based on words surrounding the target word. To generate high quality word vectors, the CBOW model can efficiently represent the target word in a context. The method to determine word vectors is shown in Figure 2. The CBOW model takes the context of each word as the input and predicts the word corresponding to the context. In Figure 2, the input layer uses one encoded vector to represent V words. V is the total number of words. The output layer is a word vector with N dimensions. The projection layer is represented by matrix *W_p_* of size VxN with each row representing a word. By learning relationships between pairs of words to update matrix *W_p_*, word vectors in the output layer can be modified and defined to represent the meaning of words.

A target word *W_t_* is the altered word at position *t* in a sequence of training words in equation (1). *W_t_* is represented by 2 words in front and 2 words after a target word.

(1) $$W_{t}=\left(w_{t-2},w_{t-1},w_{t+1},w_{t+2}\right)$$

After defining *W_t_*, word vectors can be trained using methods of hierarchical softmax and negative sampling. The hierarchical softmax method uses a Huffman tree to reduce calculation. The negative sampling method searches the maximization solution by minimizing the sampled negative instances. After training all words using these two methods, a word vector *v_t_* in equation (2) can be improved for describing the meaning of words using a matrix of target word *W_t_*. *v_t_* is the representation word vector of the target word *W_t_*.

(2) $$v_{t}=\frac{1}{\left|W_{t}\right|}\sum_{i=1}^{\left|W_{t}\right|}v_{i}$$

For testing the performance of trained word vectors for describing similarity of words in the field of a target product, results can be tested by evaluating the consistence of word vectors in the close distance for the similar meaning by the human intuition. If the word vectors cannot show a good performance for describing similarity of words in the field of a target product, more related articles of the target product will be collected to avoid overfitting. Word vectors are trained again using the updated dataset until the word vectors in the close distance are consistent with the similar meaning by the human intuition. Word vectors can then be used for comparing similarity of words in following steps.

### Collection of raw data from online customer reviews

For searching related products from online shopping websites, keywords can be defined based on main functions of products. The number of keywords can be assigned from 1 to 5 based on complexity of products. By using keywords, names and links of online products are then collected from selected online shopping websites such as Amazon, Alibaba and BestBuy using the focused web crawling method.

As the search engine for online shopping websites may find unrelated products with different functions, these unrelated products need to be removed before crawling customer reviews. Therefore, a filtering method is proposed based on similarity between keywords and names of crawled online products as follows

(3) $$\begin{array}{l} {\text{F}}_{xy}=\text{cos}(x,y)=\frac{{\vec{v}}_{x}\cdot {\vec{v}}_{y}}{\sqrt{{\vec{v}}_{x}\cdot {\vec{v}}_{x}}\sqrt{{\vec{v}}_{y}\cdot {\vec{v}}_{y}}}\;\\ \;x\in (1,2,\ldots ,\text{n})\;\;y\in (1,2,\ldots ,m) \end{array}$$

Where F*_xy_* is a similarity value between keywords and words of the name for crawled products using the cosine similarity in equation (3). Where ${\vec{v}}_{x}$ is the word vector of a keyword. ${\vec{v}}_{y}$ is the word vector of a crawled product name. n is the number of words in keywords. m is the number of words in a crawled product name.

There are n keywords in total. N*_x_* is the maximum value for the most similarity word between the *x_th_* keyword and a crawled product name in equation (4)

(4) $${\text{N}}_{x}=\text{Max}\left[F_{xy}\right]\;\;y\in (1,2,\ldots ,\text{m})\;$$

After calculating N*_x_* for n times using equation (4), values from N_1_ to N_n_ are obtained. N_Min_ in equation (5) is the minimum value from N_1_ to N_n_. N_Min_ is defined for evaluating similarity between a target product and crawled products.

(5) $${\text{N}}_{\text{Min}}=\text{Min}\left[{\text{N}}_{1}{\text{,N}}_{2}\text{,}\ldots {\text{,N}}_{\text{n}}\right]$$

N_standard_ is a value to evaluate the similarity of an online product with the target product. N_standard_ is defined in a range from 0.35 to 0.50 according to the number of keywords. If N_Min_ is lower than N_standard_, it means that the crawled online product is not similar to the target product and should be filtered. After testing all names of crawled products one by one, customer reviews in these filtered products are crawled using links of products as raw data to be used.

### Filtering unrelated words from raw data

For using raw data, the punctuation in sentences of online customer reviews is filtered by normalization. In addition, all letters in the text are converted into lowercase to avoid the influence of word cases. A natural language toolbox such as Natural Language Toolkit (NLTK) can be used to split a paragraph into individual words based on the space character in a paragraph. Parts of speech are categories to describe a word according to its syntactic functions. There are eight parts of speech including noun, pronoun, verb, adjective, adverb, preposition, conjunction, and interjection. According to characters for parts of the speech, only nouns and adjectives are selected to describe product feelings and requirements. Part-Of-Speech Tagger (POS Tagger) is a piece of software to read text for assigning parts of speech to each word. Therefore, only nouns and adjectives in customer reviews remain in raw data for defining CRs using POS Tagger.

Using nouns and adjectives, the frequency of words ${\text{F}}_{\text{w}}$ is used to filter unrelated words based on word frequency. If a word appears only a few times in a huge amount of customer reviews, these words are treated as unrelated words. The minimum frequency F_min_ of words in equation (6) is proposed to filter unrelated words to improve quality of data for the CRs definition. N_c_ is the number of customers with comments. If a word has a lower frequency than F_min_, this word is also filtered from the dataset.

(6) $${\text{F}}_{\min}=\frac{{\text{N}}_{c}}{100}$$

Some nouns and adjectives may also have little meaning for useful information to define CRs in specific functions, which can affect the clustering accuracy. Some words such as “good” and “nice” are too general to define specific CRs, so these words are also removed using an existing stop word dataset with 851 words. Some nouns such as names of products and parts may be repeated many times, but these words cannot provide any meaning for defining CRs. This kind of words will also be filtered. After filtering useless words, the left words are used for clustering in the next step.

### Clustering filtered words using the AP clustering method

The affinity propagation (AP) clustering method^20^ can cluster words into groups based on similarity of semantics in words by a mathematical distance. Each filtered word can be represented by a word vector with m characters defined in an earlier section. The distance between two words (word *i* and word *k*) in equation (7) can be measured by the distance of word vectors in equation (2)

(7) $$d(x_{i},x_{k})={\left|\left|x_{i}-x_{k}\right|\right|}_{2}=\sqrt{{\left(x_{i1}-x_{\text{k1}}\right)}^{2}+{\left(x_{i2}-x_{k2}\right)}^{2}+\cdots +{\left(x_{im}-x_{km}\right)}^{2}}$$

Responsibility matrix *R* shows the fitness of word *k* as an exemplar for word *i* in equation (8). An exemplar is the best word that explains the other words in their cluster. A cluster only has one exemplar defined by a word.

(8) R=[r(i,k)]

These responsibility values *r*(*i,k*) are determined based on a similarity function *s*(*i,k*). For searching the shortest distance between *x_i_* and *x_k_*, the squared negative Euclidian distance is defined by using word vectors in equation (9). *x_i_* is the word vector of word *i*. *x_j_* is the word vector of word *j*. The responsibility can be updated by equation (10)

(9) $$s(i,k)\text{=-}d(x_{i},x_{k})$$

(10) $$r(i,k)=s(i,k)-{\text{max}}_{k^{\prime }\neq k}\left\{a(i,k^{\prime })+s(i,k^{\prime })\right\}$$

Availability matrix *A* shows the suitable level of word *i* to choose word *k* as its exemplar. Initialization of the availabilities matrix is shown in equation (11). Where n is the number of independent words for clustering

(11) A=a(i,k)=0     i,k∈{1,2,…,n}

Availabilities matrix a(i,k) can be updated using equation (12)

(12) $$a(i,k)=\min\left\{0,r(k,k)+\sum_{i\neq i^{\prime },k\neq k^{\prime }}\left\{0,r\left(i^{\prime },k\right)\right\}\right\}$$

Self-availability a(k,k) is updated using equation (13). $i^{\prime }$ and *k* refer to the row and column of the associated matrix

(13) $$a(k,k)=\sum_{i\neq i,k\neq k}\max\left\{0,r\left(i^{\prime },k\right)\right\}$$

Criterion matrix c(i, k) in equation (14) represents that each word in the criterion matrix is simply the sum of the availability matrix and responsibility matrix at that location. *i* and *k* refer to the row and column of the associated matrix

(14) c(i,k)=a(i,k)+r(i,k)

The above steps from equations (8) to (14) are processed as a loop until the searching result remains unchanged. In order to avoid oscillation, an attenuation coefficient λ is proposed in the range of 0 to 1 based on the number of filtered words. $N_{f}$ is the number of filtered words. λ is assigned as 0.5 when $N_{f}$ is lower than 100. λ is 0.7 when $N_{f}$ is in the range of 101 to 500. λ is 0.9 when $N_{f}$ is higher than 500.

Responsibility matrix *R* and availability matrix *A* are updated for iterations using equations (15) and (16)

(15) $$r_{t+1}(i,k)=(1-\lambda )r_{t+1}(i,k)+\lambda r_{t}(i,k)$$

(16) $$a_{t+1}(i,k)=(1-\lambda )a_{t+1}(i,k)+\lambda a_{t}(i,k)$$

Exemplar *p* is defined by maximizing the sum as follows.

(17) p=arg max {a(i,k)+r(i,k)}

After iterations, the highest criterion value of each row in equation (14) is designated as the exemplar using equation (17). Rows that share the same exemplar are in the same cluster. Therefore, all the words with the same exemplar are clustered in a cluster.

### CRs definition based on exemplars in clusters

After defining exemplars in each cluster, CRs can be defined based on characters of exemplars. As words in a cluster have a similar meaning, all the words in the same cluster can be summarized as one CR. An exemplar is the best word in the group to represent the meaning of all words in a cluster.

Whether an exemplar can provide clear information for CRs is defined by the similarity between general CRs and exemplars. S*_de_*, word similarity between an exemplar and n general CRs, can be represented in equation (18) as follows. General CRs can be defined based on the field of a target product. Each general CR is described by one word.

(18) $${\text{S}}_{de}=\cos(d,e)=\frac{{\vec{v}}_{d}\cdot {\vec{v}}_{e}}{\sqrt{{\vec{v}}_{d}\cdot {\vec{v}}_{d}}\sqrt{{\vec{v}}_{e}\cdot {\vec{v}}_{e}}}\;\;\;\;e\in (0,1,\ldots \text{.}\text{.},\text{12})$$

Where ${\vec{v}}_{d}$ is a word vector of the exemplar in the *d_th_* group. *d* is the number of clusters. ${\vec{v}}_{e}$ are word vectors of general CRs. *e* is the number of general CRs.

W_standard_ is defined in the range from 0.50 to 0.65 according to the number of general CRs. W*_d_* in equation (19) is the maximum value of S*_de_*. If W*_d_* is higher than W_standard_, it means that the exemplar in the *d_th_* group has the same meaning with a general CR. Therefore, all words in the group can be defined by the exemplar in the group directly.

(19) $${\text{W}}_{d}=\mathrm{Max}\left[S_{de}\right]$$

However, additional words are required if W*_d_* is lower than W_standard_ because the exemplar cannot be directly used as a CR to provide clear design information. If an exemplar is an adjective, following nouns of the exemplar can be collected as additional words. If an exemplar is a noun, previous adjectives and nouns can be collected as additional words. The collected words with the highest frequency are selected to combine with the exemplar for defining the word phase as CRs.

Based on results of CRs, related functions and product structures can be searched for these CRs to improve customer satisfactions of the product. Algorithm 1 is for the proposed CRs definition method described in the previous sections.

**Table:** 

Algorithm 1 CRs definition
**input** frequency of word F_w_; W_standard_
**if** F_w_< F_min_
filter the word
**end if**
Filter little meaning words
Cluster filtered words to generate exemplar for each cluster
**for** i = 1 to number of exemplars
**for** j = 1 to number of general CRs
calculate word similarity S*_de_* by equation (18)
**end for**
**end for**
calculate W*_d_* = max[S*_de_*]
**if** W*_d_* >=W_standard_
the exemplar is a CR
**else if** W*_d_* < W_standard_
**if** an exemplar is an adjective
following nouns of the exemplar are combined with exemplar as a CR
**else if** an exemplar is a noun
previous adjectives and nouns are combined with exemplar as a CR
**end if**
**end if**
**output** CRs

## Case study

Two case studies are conducted to decide CRs for product design by applying the proposed method. One is the design of an upper limb rehabilitation product for professional devices, the other is the design of a mini-fridge for general consume products.

### CRs definition of the rehabilitation device

By using the proposed method, related articles were crawled and collected as a dataset for defining word vectors in the rehabilitation devices design. There are 86,57,875 sentences collected in the dataset. The number of dimensions for word vectors is defined as 300 based on the number of independent words. After filtering words with the frequency lower than 10,15,631 independent words are left in the database.

A target word *W_t_* is the altered word at position *t* shown by a sequence of training words using the CBOW method in equation (1). By using Softmax and Negative Sampling methods, a word vector *v_t_* can be trained to improve the performance for describing the meaning of words and shown in equation (2). After verifying the accuracy of trained word vectors, defined word vectors can be used to describe similarity of words for customer reviews in following steps.

After defining word vectors, raw data of upper limb rehabilitation devices were collected. Names and links of related upper limb rehabilitation devices were searched using the focused crawling method in web pages of Amazon and Alibaba. Keywords for searching in Amazon and Alibaba are defined as arm and rehabilitation based on functions of the target product. There are 211 names and links of products collected in Amazon and 76 products collected from Alibaba.

By using equations (3) to (5), 287 products were filtered into 125 products. These 125 products were used to collect raw data of customer reviews using the focused crawling method. There are 468 comments on the first product. Comments in total are 5635 for 125 products. Customer reviews found from online shopping websites are shown in Table 1. The collected data of customer online review comments of upper limb rehabilitation devices are available in an open access figshare website (https://figshare.com/articles/dataset/customer_online_reviews_of_upper_limb_rehabilitation_devices_xlsx/13298429).

**Table 1.** Customer reviews from online shopping websites.

Number	Ranking	Content of customers’ reviews
1	5 star	Sling fabric is itchy and irritates my skin. Also only works with very short arms. Even average arms won't get any wrist support.
……	……	……
468	3 star	The length of this sling from elbow to hand is perfect for me. However, there was not enough support at the elbow as the bottom of the sling slopes.
……	……	……
5635	2 star	Second time I've had shoulder surgery this year. The nylon sling the hospital provides is harsh on the neck and generally uncomfortable.

Punctuations in the reviews were filtered and sentences were transferred into individual words based on the space character. Words of customer reviews were filtered based on parts of speech using Stanford Log-linear POS Tagger and stop words introduced in an earlier section. The frequency is defined as 56 using equation (6). Words with the frequency less than 56 times are filtered in the dataset.

After filtering words, there are 79 words left. They are then clustered by the AP clustering method. The similarity of two words is measured by the distance of the word vectors using equation (7). Responsibility matrix *R* is defined using equations (8) to (10). Availability matrix *A* is determined using equations (11) to (13). Criterion matrix c(i, k) measures the sum of the availability matrix and responsibility matrix using equation (14).

In order to avoid oscillation, responsibility matrix *R* and availability matrix *A* are updated for iteration using equations (15) and (16). The result of clustering groups is shown in the second column in Table 2. The exemplar of each group is defined by maximizing sum c(*i*,*k*) using equation (17) and shown in the third column in Table 2.

**Table 2.** Word clusters by the AP clustering method.

	Words in each cluster	Words of exemplars	Number of words in each cluster
1	Flexibility, posture, adaptability, adjustable, adjustment, flexible, wear, tight	Flexible	8
2	Safe, safety, injury, dangerous, health, unsafe, protective, harmful, injured, pain, uncontrolled, wounded	Safety	12
3	Light, heavy, lighter, weight, heavier, lightweight, weights	Lightweight	7
4	Cheap, cheaper, expensive, affordable, money, price, discount, worth	Price	8
5	Chemical, odors, odor, smell, disgusting, stimulating, mold,	Smell	7
6	Supporting, strengthening, supports, assistance, support, supportive, aid, handle, help	Support	9
7	quality, durability, toughness, strength, durable, sturdy	Durable	6
8	Comfort, comfortable, uncomfortable, comfy, cozy	Comfort	5
9	Shaking, fastening, swinging, fastened, loose, shake,	Fastening	6
10	size, large, larger, smaller, small, longer, short, fit, length, thickness, ranges	Size	11
Total number of words			79

General CRs are defined by summarizing the existing literature of rehabilitation device design.^21^ There are 12 general CRs including price, comfort, brake, durability, safety, sustainability, portable, adjustable, maintenance, operation, assembly and weight.

For deciding whether an exemplar can be used as a CR directly, words of exemplars are tested using equations (18) and (19). Based on the number of general CRs, W_standard_ is defined as 0.6. If W*_d_* is higher than 0.6, CRs are defined using exemplars directly. Meanwhile, 5 exemplars including flexible, smell, support, fastening and size cannot be used as CRs directly because W_1_, W_5_, W_6_, W_9_ and W_10_ in the second column of Table 3 are lower than 0.6. The final CRs are defined in Table 3.

**Table 3.** CRs definition by exemplar.

Exemplar	W in equation (19)	Top 3 words in front of exemplar			Final CRs
		1	2	3	
1. Flexible	0.27	Wear	Movement	Arm	CR.1 flexible wear
2. Safety	1.0	None			CR.2 safety
3. Lightweight	0.65	None			CR.3 lightweight
4. Price	1.0	None			CR.4 price
5. Smell	0.23	No	Chemical	Terrible	CR.5 no smell
6. Support	0.29	Arm	Shoulder	Elbow	CR.6 arm support
7. Durable	0.75	None			CR.7 durable
8. Comfort	1.0	None			CR.8 comfort
9. Fastening	0.25	Elbow	Forearm	Elbows	CR.9 elbow fastening
10. Size	0.28	Forearm	Elbow	Wrist	CR.10 forearm size

The last column in Table 3 is CRs defined by exemplar and top 3 words using the proposed method. Based on the results of CRs, related functions and specifications can be decided for these CRs to improve customer satisfactions for the devices.

### Verification by online reviews analysis and experiment of rehabilitation devices

For verifying advantages of the proposed method, CRs defined by existing survey methods are compared to CRs from the proposed method. The literature^22^ used the existing customer survey method to collect customer comments and defined CRs of the upper limb rehabilitation device by designers. The results are shown in the second column in Table 4. CRs defined by the proposed method are shown in the last column of Table 4. Results of CRs comparisons by the existing survey method and proposed method are also shown in Table 4.

**Table 4.** CRs comparison by the existing method and proposed method.

Number	CRs by existing methods	Comment for CRs by existing method and proposed method	CRs by proposed method
1	Adaptability	CR.1 and CR.2 in the existing method is combined as a CR.	CR.1 flexible wear
2	Wearable		
3	Safety	Same CRs 2-4 and CRs 6-8 in the existing method and proposed method.	CR.2 safety
4	Lightweight		CR.3 lightweight
5	Price		CR.4 price
6	Support the arm		CR.6 arm support
7	Durability		CR.7 durable
8	Comfortable		CR.8 comfort
9	Easy operation	These 3 CRs in the existing method are removed from proposed method.	None
10	Portability		
11	Easy to store		
12	None	CR.5, CR.9 and CR. 10 in the proposed method are 3 CRs ignored by existing method.	CR.5 no smell
13			CR.9 elbow fastening
14			CR.10 forearm size

Comparing with the existing method, the proposed method adds 3 CRs including CR.5 no smell, CR.9 elbow fastening and CR.10 forearm size. The proposed method does not have 3 CRs of easy operation, portability and easy to store because customers do not care about the performance of products related to these 3 CRs. In addition, 2 CRs with similar meaning in the existing method are combined into one CR in the proposed method. Adaptability and wearable in the existing method are combined as one CR called flexible wear in the proposed method.

For evaluating the accuracy of CRs defined by the proposed method, online reviews from the product with the lowest rating are analysed. A product called Soles Hinged Elbow Brace for Injury Recovery has the lowest average rating 2.9. There are 167 unsatisfied customer reviews for the product in total. Percentages of unsatisfied customer reviews for each related CR are shown in Table 5.

**Table 5.** Unsatisfied reviews for the product with lowest rating.

Existing method		Proposed method	
CRs	Rating	CRs	Rating
1. Adaptability	3.8%	CR.1 flexible wear	5.9%
2. Wearable	2.2%		
3. Safety	5.5%	CR.2 safety	5.5%
4. Lightweight	4.5%	CR.3 lightweight	4.5%
5. Price	0.6%	CR.4 price	0.6%
6. Support the arm	10.9%	CR.5 no smell	12.0%
7. Durability	7.1%	CR.6 arm support	10.9%
8. Comfortable	10.0%	CR.7 durable	7.1%
9. Easy operation	1.0%	CR.8 comfort	10.1%
10. Portability	0.6%	CR.9 elbow fastening	18.2%
11. Easy to store	0%	CR.10 forearm size	18.8%
Not included	53.8%	Not included	6.3%

In Table 5, there are 53.8% of unsatisfied comments in the product with the lowest rating that are not included in CRs in the existing method, because two important CRs of CR.9 elbow fastening and CR.10 forearm size are missed. In addition, there are very few unsatisfied comments for easy operation, portability and easy to store. Some unnecessary and unimportant CRs are included in the exiting method, which can affect accuracy of CRs. In the proposed method, only 6.3% of customer reviews cannot be found. Therefore, the proposed CRs method can find most of necessary CRs accurately for the product with the lowest rating. Based on the results in Tables 5, the proposed method has shown better performance in the CRs definition accurately and efficiently compared to the existing method.

**Figure 3.**
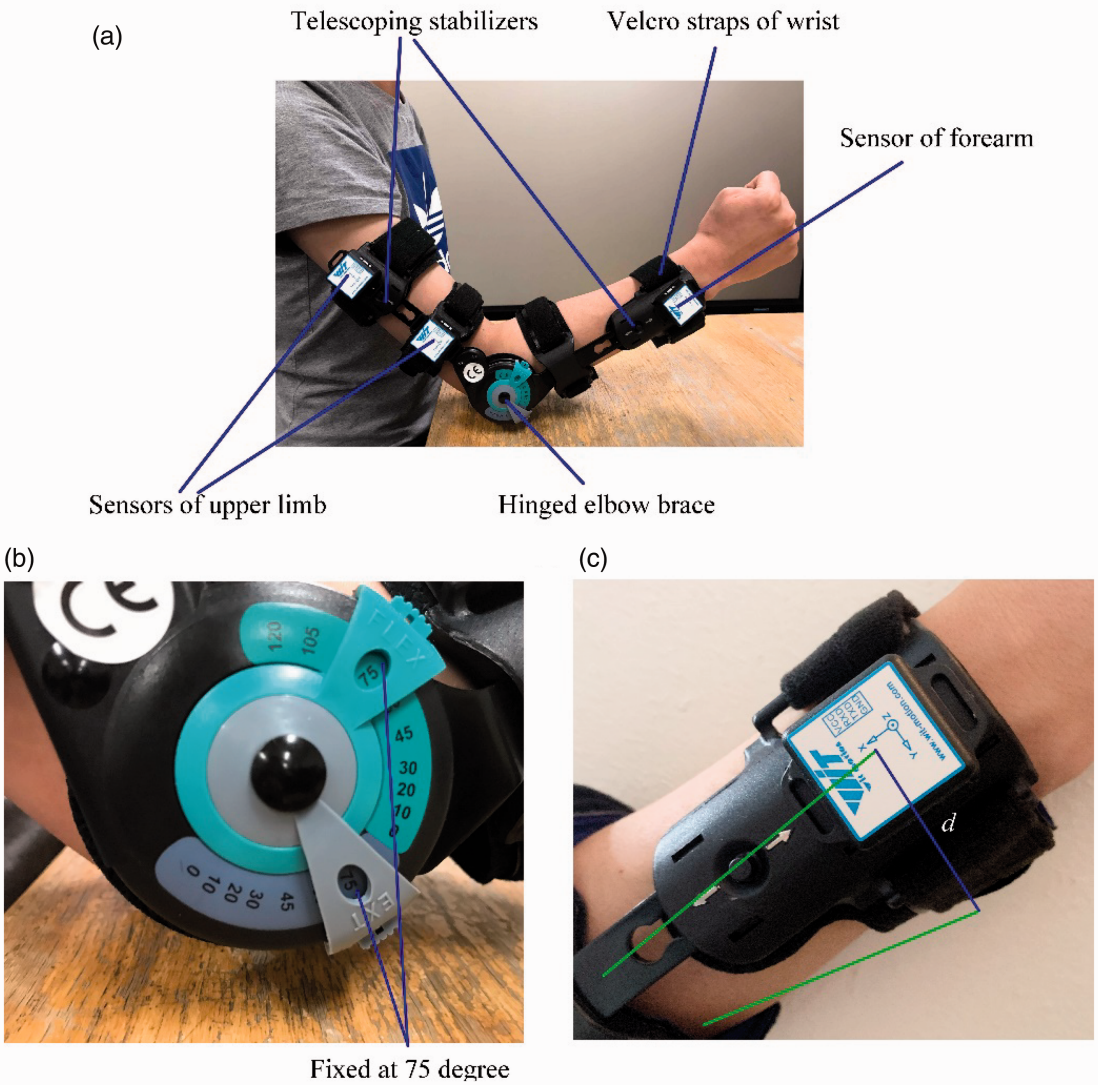
(a) Upper limb rehabilitation device; (b) hinged elbow brace; (c) displacement of wrist.

The product with the lowest rating called Soles Hinged Elbow Brace for Injury Recovery is shown in Figure 3(a). Specifications of tested rehabilitation devices in the third column of Table 6 are from the product manuals. Required specifications for customers in the fourth column of Table 6 are defined by an average value of specifications from the online collected products.

**Table 6.** Specifications and testing results of upper limb rehabilitation devices.

Product specifications	Specifications of tested rehabilitation device	Required specifications for customers	Related CRs	Meet related CRs or not
Frame material	Plastic with styrene	Health	CR.5	No
Cover material	Cotton	Comfort	CR.8	Yes
Whole size	500*80*90 mm	None	CR.1	Yes
Length of forearm	180–235 mm	170–270 mm	CR.10	No
Length of upper arm	170–230 mm	170–220 mm	CR.6&7	Yes
Elbow angle	0–120 degree	0–120 degree	CR.2	Yes
Diameter of forearm	50–180 mm	50–150 mm	CR.1&7CR.1&7	Yes
Diameter of upper arm	50–180 mm	50–150 mm		Yes
Price	115 US Dollar	Less than 300 US Dollar	CR.4	Yes
Weight	0.53 kg	Lower than 3 kg	CR. 3	Yes
Displacement *d* of wrist movementAngle θ of wrist movement	15.3 mm	0-2.0 mm	CR.9	No
	3.5 degree	0–1.0 degree	CR.9	No

As all specifications of the product are not related to CR.9, an experiment is conducted to test CR.9. Figure 3(a) shows structures of the upper limb rehabilitation device. It can control elbow range of the motion for improving the stability and recovery. “Easy Hinge” can be used to control elbow range of the motion. Along with Velcro straps, each elbow brace features telescoping stabilizers to help patients finding the right stability and comfort level. The device can be used for the arm injuries recovery such as dislocations and fractures. In Figure 3(b), the hinged elbow brace should be fixed at 75° to help patients with fractures or injuries to fix their arms for recovery. Two wireless motion sensors of upper arms are used to ensure that upper arms are fixed. One wireless motion sensor of forearm is used to measure displacement *d* of wrist in Figure 3(c) for testing the stability of the device. The motion sensor provides coordinate positions in x-y-z directions. The accuracy of the motion sensors is 0.01 mm. The acquisition frequency is 50 Hz. Based on two collected coordinate locations, the displacement of wrist *d* can be decided.

In Figure 3(c), if *d* is very low, it means that the device can meet CR.9 and the device does not have any problem for loose elbow structure. Movement angle θ of the hinged elbow brace can be measured by equation (20). Displacement *d* of wrist is defined by multiplying velocity of motion sensor and movement time. *l* is length from elbow to sensor of forearm.

(20) $$\theta =\frac{360{}^{\circ}d}{2\pi l}$$

Specifications and testing results for the upper limb rehabilitation device are shown in Table 6. Results show that the device cannot meet CR.5, CR.9 and CR.10. CR.5 no smell cannot be met in the device because the frame material is plastic with styrene which has a terrible smell. CR. 9 elbow fastening cannot be met because the maximum and average displacement of the fixed elbow structure are too high. CR.10 forearm size cannot be met because the device cannot reach the wrist and provide wrist support for 30% patients whose forearm is longer than 235 mm. These 3 CRs are ignored in the existing design of the product with the lowest rating. However, these 3 CRs can be found in the proposed method. If a product can be designed by using CRs defined by the proposed method, these 3 CRs can be met in the product, which can improve customer satisfactions significantly.

According to specifications and testing results in Table 6, designers ignored these necessary CRs including CR.5 no smell, CR.9 elbow fastening and CR.10 forearm size in the design process. In the existing customer survey methods such as focus groups survey and web-based survey methods, CRs can only be defined based on the customer survey or designers experience. As the limited number of surveyed customers, CRs cannot be decided accurately. For example, the CR.9 and CR.10 are difficult to be decided because some of their characters require feedbacks or comments from a large number of customers. The existing method can only consider a part of characters of the rehabilitation device. Based on the limited number of questionnaires from customer surveys, CRs cannot be defined accurately using the existing survey method.

The proposed method can collect feedbacks or comments from a huge number of customers, for example, comments of 5653 customers were used by the focused crawling method. Therefore, the proposed CRs definition method can find the most common and important word from user comments to determine CRs accurately and efficiently.

### Comparison of the state-of-the-art method and proposed method

For further verifying advantages of the proposed method, a state-of-the-art CRs definition method, called the affinity diagram method,^23^ is compared with the proposed method. Customer comments collected by interviews with patients using questionnaires are summarized in a dendrogram with four levels to define CRs based on the affinity diagram method. Seven CRs including control, adapting, reliability, assistance, self-motivation, portability and size are defined for the upper limb rehabilitation device.

Seven CRs defined by the affinity diagram method^23^ cannot decide all important CRs for rehabilitation devices accurately because some necessary CRs such as safety, comfort and elbow fastening are ignored. As the affinity diagram method uses customer comments from limited numbers of interviewed patients, these patients cannot provide enough comments for requirements in different aspects. For example, they missed comfort as a CR. In addition, the dendrogram can only divide customer comments into three or four levels by the hierarchical clustering algorithm, which cannot define the most representative words as CRs. For instance, the CR assistance cannot provide a clear meaning for designers to decide related functions and structures to meet the CR.

Using the proposed method, comments are automatically collected from 5635 customers in the product shopping webpage. All the important CRs of the device can be decided to match the customer comments easily. CRs such as elbow fastening and no smell are defined by the proposed method for designers to improve the design of devices. In addition, the proposed method can combine customer comments for exemplars to define CRs using the AP clustering method, which can summarize the most representative words such as safety and comfort as CRs to cover different requirements. Therefore, the proposed method can define CRs accurately and efficiently.

### CRs definition of the mini-fridge

Customer online comments of mini-fridges were collected to define CRs. By using equations (3) to (5), 53 products were collected for raw data of customer reviews using the focused crawling method. The exemplar of each group is defined in the first column of Supplementary Table 7 using equations (6) to (17). The final CRs are defined in the last column of Supplementary Table 7 based on equations (18) and (19).

The last column in Supplementary Table 7 is the final CRs of mini-fridges defined by the proposed method. Based on results of CRs, related functions and specifications can be decided for meeting these CRs to improve customer satisfactions of mini-fridges.

The collected data are available in an open access figshare website (https://figshare.com/articles/dataset/customer_online_reviews_of_mini-fridges_xlsx/13298438).

### Verification of online reviews analysis and experiment of the mini-fridge

The most popular product called the AstroAI mini-fridge with 136 unsatisfied customer reviews is used to verify the proposed method for defining CRs of the mini-fridge. Percentages of unsatisfied customer reviews for each related CR of the mini-fridge are shown in Supplementary Table 8.

In Supplementary Table 8, 30.4% of unsatisfied comments in the product with the lowest rating are not included in CRs in the existing method because two important CRs, CR.8 stable temperature and CR.11 no leaking, are missed. In the proposed method, only 4.1% of customer reviews cannot be found. Therefore, the proposed CRs method can find most of necessary CRs of a product accurately.

**Figure 4.**
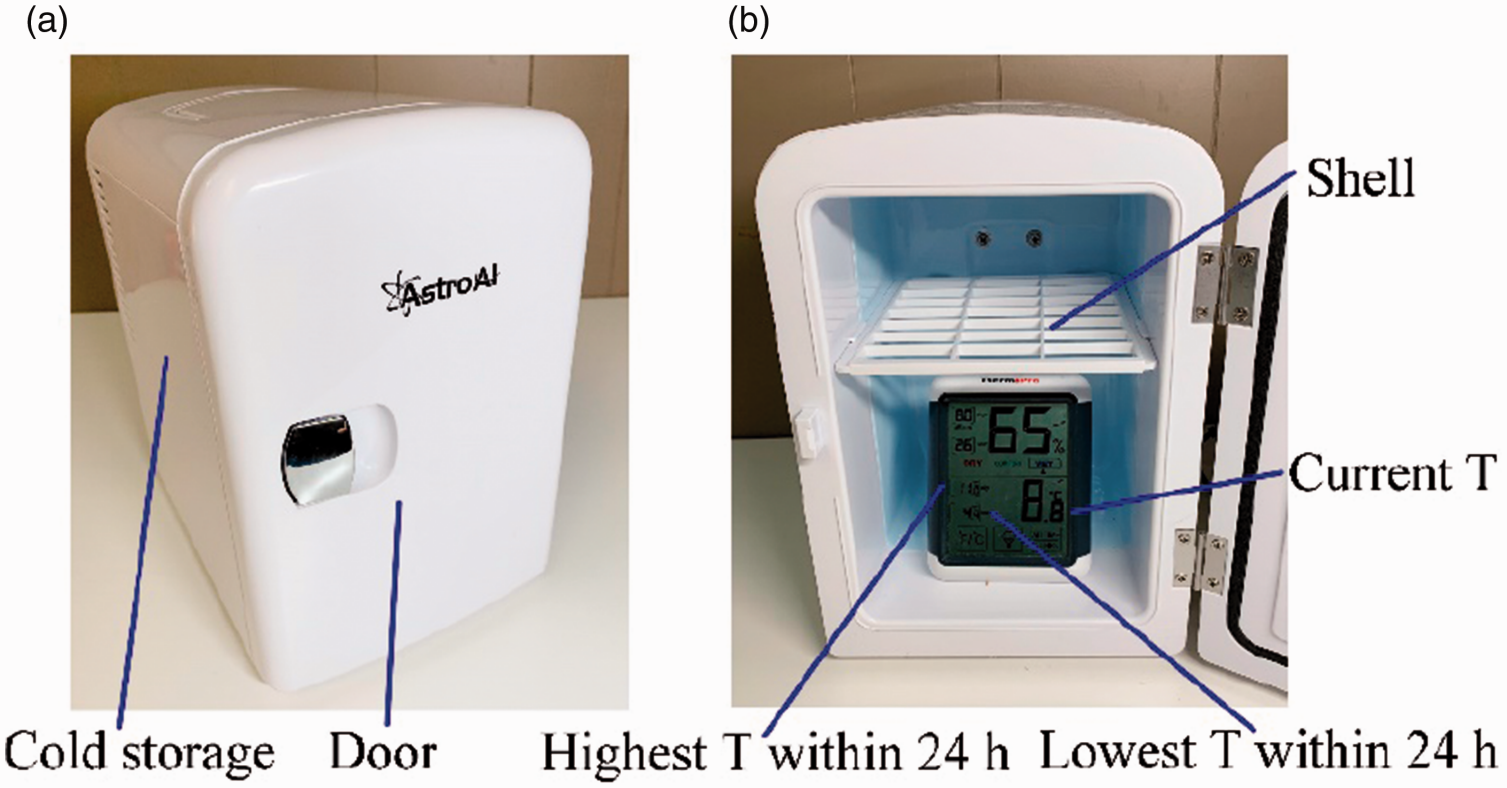
(a) The mini-fridge; (b) temperature testing.

For further verifying the performance of mini-fridges, the AstroAI mini-fridge shown in Figure 4(a) was used as an example for the detail analysis, its temperature (T) was tested for 24 hours as shown in Figure 4(b).

Specifications and testing results of the mini-fridge are shown in Supplementary Table 9. Results show that this mini-fridge cannot meet CR.8 and CR.11. CR.8 stable temperature cannot be met because the temperature sensor is not sensitive enough to maintain the temperature at the stable range. CR.11 no leaking cannot be met because the sealing strip around the door cannot fully cover the door and there is not a magnet for closing the door tightly.

In the existing methods for defining CRs of products, designers normally determine CRs based on their experience to save time and cost in the product design process. Designers can define general CRs such as cost and durability successfully based on the experience. However, some important and detailed CRs of a product can easily be ignored using the existing methods because some CRs can only be identified by reviewing comments from different customers who have used the product. In fact, the proposed method can define all CRs accurately using the AP clustering method based on a huge amount of customer reviews collected online. For example, CR.8 and CR.11 ignored by the existing method are found using the proposed method. Therefore, the proposed method has a better performance for defining CRs of products.

## Conclusions and future work

This research proposed a new CR definition method based on the data crawling and AP clustering method. Word vectors were determined using the CBOW method. After filtering online customer reviews using parts of speech and frequency of words, filtered words were clustered into groups by the AP clustering method. CRs were then defined by exemplars in each group and similarity between exemplars and general CRs. According to the case study and experiment of the upper limb rehabilitation device, advantages of the proposed method are verified for defining CRs effectively and accurately from online customer reviews.

Main contributions and innovations of this paper are summarized as follows. (1) Efficiency of defining CRs is improved by automatically collecting data from the product shopping webpage using the focused crawling method. (2) Accuracy of defining CRs is improved by combining different online comments from customers to find an exemplar to represent the meaning of all the words in a cluster using the AP clustering method. (3) The proposed method can determine CRs to best match the consumer interests by using crawled online customer reviews.

The further work will define weights of CRs based on meaning of a whole sentence using customer reviews. The meaning of the whole sentence can be measured by word vectors to provide more information for defining weights of CRs. Structures of products can be improved by defining relationships between CRs and engineering characters using data mining such as methods of classification and association rule learning.

## Supplemental Material

sj-pdf-1-pie-10.1177_09544089211001776 - Supplemental material for Definition of customer requirements in big data using word vectors and affinity propagation clustering Click here for additional data file. Supplemental material, sj-pdf-1-pie-10.1177_09544089211001776 for Definition of customer requirements in big data using word vectors and affinity propagation clustering by Yanlin Shi and Qingjin Peng in Proceedings of the Institution of Mechanical Engineers, Part E: Journal of Process Mechanical Engineering
