# Supplementary material for: Definition of customer requirements in big data using word vectors and affinity propagation clustering
Source: Proc Inst Mech Eng E J Process Mech Eng. 2021 Mar 16;235(5):1279–91. doi: 10.1177/09544089211001776 (PMC8494268; doi:10.1177/09544089211001776)
Supplement: sj-pdf-1-pie-10.1177_09544089211001776 - Supplemental material for Definition of customer requirements in big data using word vectors and affinity propagation clustering [file sj-pdf-1-pie-10.1177_09544089211001776.pdf]

Supplementary Tables

Supplementary Table 7 CRs definition by exemplars for mini-fridges

Exemplar	W in Eq. (19)	Top 3 words in front of exemplar			Final CRs
		1	2	3	
1. durable	0.76				CR.1 durable
2. cooling	0.35	speed	mode	device	CR.2 cooling speed
3. quiet	0.87				CR.3 quiet
4. frost	0.28	no	fridge	back	CR.4 no frost
5. smell	0.33	no	pure	special	CR.5 no smell
6. power	0.37	consumption	option	function	CR.6 power consumption
7. price	1.0				CR.7 price
8. temperature	0.21	stable	sensitive	room	CR.8 stable temperature
9. cleaning	0.87	easy	quick	suitable	CR.9 easy cleaning
10. lightweight	1.0				CR.10 lightweight
11. leaking	0.32	no	water	constant	CR.11 no leaking

Supplementary Table 8 Percentages of unsatisfied customer reviews for the AstroAI mini-fridge

Existing method		Proposed method	
CRs	Rating	CRs	Rating
1. durability	12.2%	CR.1 durable	12.2%
2. quick freezing	7.5%	CR.2 cooling speed	7.5%
3. less noise	15.3%	CR.3 quiet	15.3%
4. no frost	11.2%	CR.4 no frost	11.2%
5. no smell	6.3%	CR.5 no smell	6.3%
6. less energy consumption	3.2%	CR.6 power consumption	3.2%
7. low price	5.1%	CR.7 price	5.1%
8. adjustable shell	1.5%	CR.8 stable temperature	16.3%
9. easy to clean	2.2%	CR.9 easy cleaning	2.2%
10. easy to carry	5.1%	CR.10 lightweight	5.1%
Not included	30.4%	CR.11 no leaking	11.5%
		Not included	4.1%

Supplementary Table 9 Specifications and testing results of the mini-fridge

		Result	Required specifications for customers	Related to proposed CRs	Meet or not
Product specifications	Energy consumption	0.2 KWh/day	0.5 KWh/day	CR.6	Yes
	Noise level	35 dB	40 dB	CR.3	Yes
	Required cooling time	3 hours	5 hours	CR.2	Yes
	Warranty time	3 years	2 years	CR.1	Yes
	Price	60 US Dollar	Lower than $100	CR.7	Yes
	Material	Plastic	toxic free and no smell	CR.5	Yes
	Weight	0.53 Kg	Lower than 2 kg	CR. 10	Yes
Product test	Rage of temperature (°C)	4.4-11.0°C	Less than 3°C change	CR.8	No
	Gap for door	1.7 mm	Lower than 0.5 mm	CR.11	No
	Water in bottom per 24h	3 mL	0-5mL	CR.4	Yes
